# Vertebral Fracture in an Elderly Golfer

**DOI:** 10.7759/cureus.27463

**Published:** 2022-07-29

**Authors:** Masahiro Kawanishi, Hidekazu Tanaka, Yutaka Ito, Kunio Yokoyama, Makoto Yamada

**Affiliations:** 1 Neurosurgery, Takeda General Hospital, Kyoto, JPN

**Keywords:** elderly, swing, return to sport, vertebral fracture, golfer

## Abstract

Golf is one of the most popular sports among seniors. Here, we report the case of a 76-year-old woman who developed a vertebral fracture while playing golf. The patient had been suffering from leg pain for several years but developed sudden back pain after her golf swing. Because magnetic resonance imaging demonstrated a new vertebral fracture of the L1 vertebral body and canal stenosis at the L4/5 level, she successfully underwent L1 vertebroplasty and L4/5 decompression. For older golfers, a classical swing that twists the pelvis and shoulders at the same time may be recommended.

## Introduction

While golf is one of the most popular sports among seniors [1], there have been reports of various secondary spine injuries while playing golf [2]. Here, we report the case of a 76-year-old woman who developed a vertebral fracture while playing golf. In addition, we review the various effects of golf on the spine and the time to return to play after surgery.

## Case presentation

Clinical presentation

**Figure 1:**
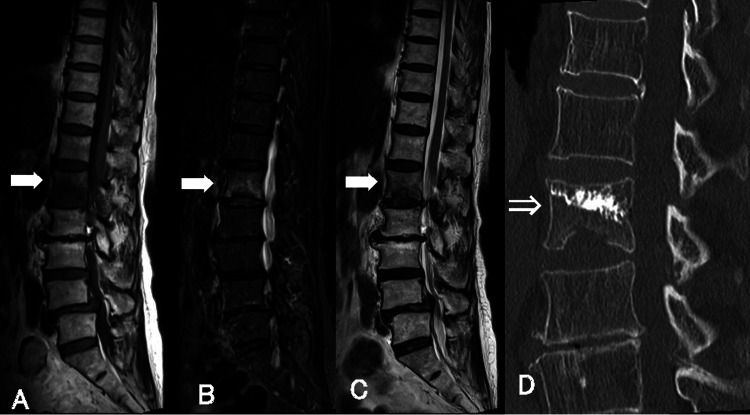
Preoperative MRI and CT after vertebroplasty. A-C: Thoracolumbar MRI showing a new vertebral fracture of the L1 vertebral body (white-painted arrow). A: T1WI; B: STIR; C: T2WI. D: Thoracolumbar CT showing augmentation of polymethyl methacrylate (white-open arrow). MRI: magnetic resonance imaging; CT: computed tomography; WI: weighted imaging; STIR: short-TI inversion recovery

A 76-year-old woman who had played golf for more than 40 years suddenly noted severe back pain after swinging. Although she had recently developed leg pain and claudication, she kept playing golf like the modern golf swing. She visited our clinic a few days later complaining of typical back pain on changing position and bilateral leg pain without weakness. Magnetic resonance imaging (MRI) showed a new fracture of the L1 vertebral body and lumbar spinal canal stenosis caused by grade 1 spondylolisthesis and lumbar disc herniation; however, the instability was not recognized symptomatically or on imaging at the L4/5 level (Figure 1, Panel A-C).

Surgery

First, vertebroplasty was performed on the L1 lumbar vertebra (Figure 1, Panel D). We performed only decompression without instrumentation or fixation as the L4/5 lumbar vertebrae were considered mechanically stable along with degenerative spondylolisthesis and potential osteoporosis. Her back pain and leg pain were completely resolved, and she returned to golf about two months after discharge.

## Discussion

Recently, golf has become a popular sport among the elderly, with a very high percentage of elderly golfers. Golf is sometimes recommended as one of the best sports to prevent frailty in the elderly [3].

Golf injuries

**Figure 2:**
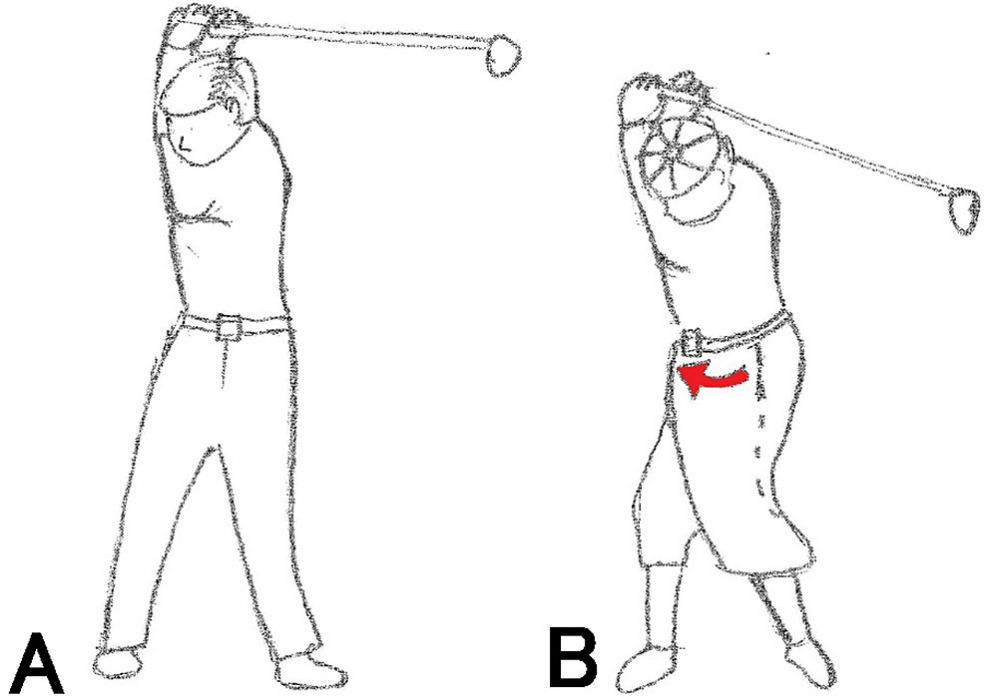
Modern swing (A) and classic swing (B). The pelvis and shoulders are largely rotated in the same direction, as shown by the red arrow in the classic swing (B), whereas the pelvis is rotated as little as possible and the shoulders are rotated in the modern swing (A).

Golfers have various musculoskeletal disorders or golf swing-related fractures, including stress fractures caused by repeated bone shocks and fractures caused by rapid twisting [4,5]. Stress fractures occur in the ribs, ulnar diaphysis, the spinal process of vertebrae, sternum, and hook of hamate [2]. There are two types of golf swings, namely, the classic swing, in which the trunk is turned without twisting and the pelvis is turned with the shoulder, and the modern swing, in which the shoulder is turned without turning the pelvis as much as possible and a twisting force is applied when returning [4]. Fractures of the spinous process of the lower cervical and upper thoracic vertebrae due to rapid torsion of the cervicothoracic vertebrae are well known as Clay-Shoveler’s fracture [6]; however, there has been only one previous report on vertebral fractures in golfers [7]. The compression fracture of the first lumbar vertebra in our case occurred in one swing, and it is presumed that it was caused by the excessive load caused by the twist to the thoracolumbar junction. Classic golf swing mainly focuses on hip rotation. However, in modern swings, instead of hip rotation, which emphasizes shoulder rotation, it increases the torsional load on the lumbar spine and involves hyperextension to get maximal club head velocity and driving distance (Figure 2).

It is expected that the number of elderly people playing golf will continue to increase in the future. Elderly players can be encouraged to use the classic style of turning the pelvis with the trunk instead of twisting, depending on the situation.

Return to golf after lumbar spine surgery

There are a few reports of a low level of evidence (3 or less) regarding the number of days after lumbar spinal surgery to return to golf [1,8-11]. If golf is considered a non-contact sport, the reported return time for non-fusion surgeries, such as lumbar disc herniation surgery, is four to eight weeks [4]. On the other hand, when lumbar fusion is performed, the time to return is reported to be four to eight months [1,8,10,11] (Table1).

**Table 1:** Period of return to golf after lumbar spine surgery. RTG: return to golf; NA: not applicable

	Abla et al. 2011 [8]	Shifflett et al. 2017 [10]	Jain et al. 2020 [1]	Zuckerman et al. 2021 [11]
	Laminectomy			Fusion
RTG	Discectomy: 4–8 weeks	Fusion: 6 months	Fusion: 8.6 months	Degenerative: 4.3 months
	Fusion: 6 months			Deformity: 9.7 months
Study design	Survey of surgeon’s opinion	Survey	Survey	Case series
Sample size	523 responded	34	13	6
Mean age	NA	57	64	60

The return to golf was determined to be eight weeks in this case because no intervertebral fusion was performed.

## Conclusions

For elderly golfers, a classical swing that twists the pelvis and shoulders at the same time may be recommended. The timing of return to golf may be appropriate at about eight weeks for non-fusion surgery and six months for fusion surgery based on the low-level evidence.
